# Diminished function of cytotoxic T- and NK- cells in severe alcohol-associated hepatitis

**DOI:** 10.20517/mtod.2022.13

**Published:** 2022-10-25

**Authors:** Adam Kim, Christina K. Cajigas-Du Ross, Jaividhya Dasarathy, Annette Bellar, David Streem, Nicole Welch, Srinivasan Dasarathy, Laura E. Nagy

**Affiliations:** 1 Northern Ohio Alcohol Center, Department of Inflammation and Immunity, Cleveland Clinic, Cleveland, OH 44195, USA.; 2 Department of Family Medicine, MetroHealth, Cleveland, OH 44195, USA.; 3 Lutheran Hospital, Cleveland Clinic, Cleveland, OH 44195, USA.; 4 Department of Molecular Medicine, Case Western Reserve University, Cleveland, OH 44195, USA.; 5 Department of Gastroenterology and Hepatology, Cleveland Clinic, Cleveland, OH 44195, USA.

**Keywords:** Alcohol-associate Hepatitis, inflammation, NK cells, cytolytic granules, single-cell RNA-seq

## Abstract

**Aim:**

Metabolic liver diseases, including alcohol- and non-alcoholic fatty liver diseases (ALD/NAFLD), are characterized by inflammation and decreased ability to prevent infections. Patients with severe alcohol-associated hepatitis (sAH) are particularly susceptible to infections while undergoing treatment with steroids. Understanding the immunological mechanisms for these responses is critical to managing the treatment of patients with metabolic liver diseases. Cytotoxic NK cells and CD8 T cells, using cytolytic granules, serve an important immunological role by killing infected cells, including monocytes. However, patients with sAH have dysfunctional NK cells, which cannot kill target cells, though the mechanism is unknown.

**Method:**

We performed an exploratory study using single-cell RNA-seq (scRNA-seq) (*n* = 4) and multi-panel intracellular flow cytometry (*n* = 7–8 for all patient groups) on PBMCs isolated from patients with sAH and healthy controls (HC).

**Results:**

ScRNA-seq revealed receptors in NK cells and CD8 T cells required for cytotoxic cell recognition of activated monocytes were downregulated in patients with sAH compared to healthy controls. Granulysin was the most downregulated gene in both NK cells and effector CD8 T cells. In NK cells from HC, expression of granulysin, perforin, and granzymes A and B was highly correlated; however, in sAH, these genes lost coordinate expression, indicative of dysfunctional cytolytic granule formation. Finally, the expression of cytolytic granule proteins in NK cells was decreased from sAH, indicating reduced cytolytic granules.

**Conclusion:**

Together, these results suggest a loss of cytotoxic cell function in PBMCs from sAH that may contribute to a decreased ability to communicate with other immune cells, such as monocytes, and prevent the killing of infected cells, thus increasing the risk of infection.

## INTRODUCTION

Chronic alcohol intake and excessive calorie consumption are two major factors in the development of fatty liver diseases, including alcohol- and non-alcoholic fatty liver diseases (ALD/NAFLD). Metabolic liver diseases are characterized by inflammation and decreased ability to prevent infections^[1]^. These immune dysfunctions can become life-threatening, particularly in patients with severe alcohol-associated hepatitis (sAH) while undergoing treatment with steroids^[1]^, currently the only approved therapeutic strategy for treating sAH. The immune dysfunctions in ALD and NAFLD are similar in many ways^[1]^, and there is a growing appreciation that alcohol consumption combined with obesity synergistically impacts immune functions regulating bacterial and viral infections^[2–4]^. As alcohol can contribute significantly to metabolic dysfunction in NAFLD and obesity can complicate ALD, clinicians and researchers need better tools and outcomes for studying how these different metabolic disorders lead to specific cellular, immunological, and organ tissue damage^[5]^.

Chronic alcohol consumption is associated with defects in immunometabolism, leading to dysfunctional innate and adaptive immune responses^[6–8]^. The immune system becomes hyper-inflammatory but also less able to prevent and eliminate microbial infections^[8]^. While this is generally true for chronic heavy drinkers and patients with ALD, different stages of ALD may lead to variable cell-specific phenotypes and deficiencies. Cytotoxic immune cells (NK cells and CD8 T cells) play a critical role in regulating immune cells during infection; regardless of stage, their function is reduced in patients with ALD, though the mechanisms of such dysfunction are currently unknown^[9–12]^.

**Figure 1.**
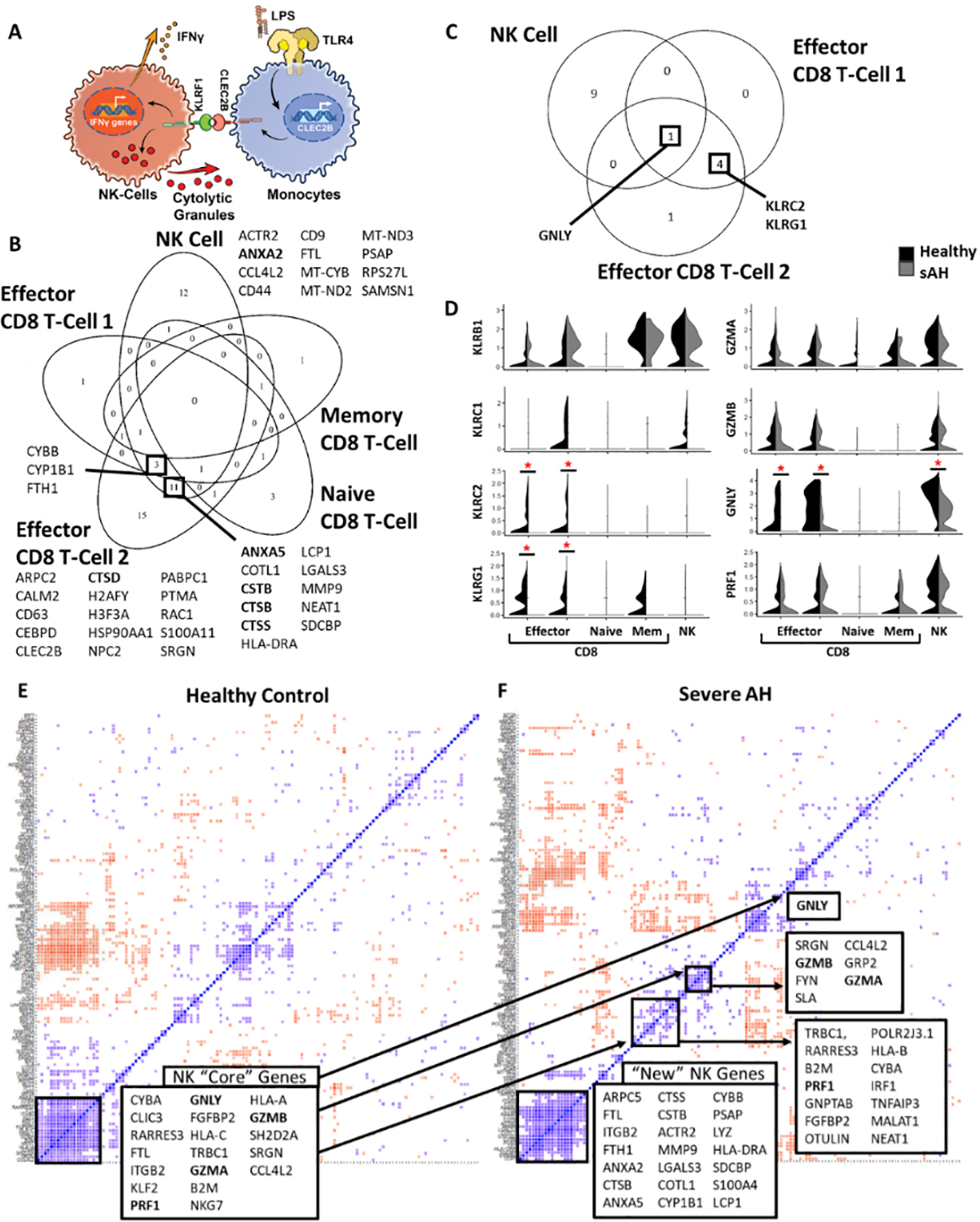
scRNA-seq reveals genes differentially expressed in cytotoxic cells between sAH and HC. (A) Schematic showing how NK cells recognize activated target cells and how NK cells respond. (B) Venn Diagram showing genes commonly upregulated in sAH between NK cells and four subtypes of CD8 T cells: Naïve, Memory, and two distinct clusters of Effector cells. (C) Venn Diagram showing genes commonly downregulated in sAH between NK cells and effector CD8 T cells. (D) Violin plots showing expression of major C-type lectin receptors (Left) and components of cytolytic granules (Right). * = *P* < 0.05. (E and F) Correlation analysis to see how genes in NK cells are coordinately expressed at baseline for healthy control (E) and sAH (F). Blue squares indicate genes with highly correlated expression, while red squares indicate anti-correlated expression (see “METHODS”). sAH: Severe alcohol-associated hepatitis; HC: healthy controls.

Cytotoxic immune cells have two primary functions: kill infected, stressed, or malignant cells and regulate other cells via interferon gamma (IFNγ) signaling [Figure 1A]^[11]^. Cytotoxic cells perform these functions by binding target cells and then killing them via exocytosis of cytolytic granules. NK cells, part of the innate immune system, recognize target cells with paired cognate C-type lectin receptors (CLRs) and the Immunoglobulin-like Leukocyte Receptors^[11]^. For example, monocytes express CLEC2B (AICL, mice) in response to TLR signaling, which is recognized by KLRF1 (NKp80, mice)^[13]^. CD8 T cells, part of the adaptive immune system, recognize T-cell receptor ligands displayed on the surface of target cells. Upon binding, both cells release cytolytic granules, which consist of perforin (PRF1), granzymes A and B (GZMA, GZMB), and in humans, granulysin (GNLY)^[14]^. After endocytosis, perforin creates pores in membranes allowing the release of granzymes, a family of proteases, and the antimicrobial peptide granulysin, which induce apoptosis^[15]^. Only mature cytotoxic cells express cytolytic granules, with genes involved in cytolysis turned on in a step-wise manner during maturation^[16]^.

The impact of alcohol consumption and its metabolic consequences on cytotoxic T-cells is controversial^[17]^. One study found reduced cytolytic function in NK cells in patients with alcoholic cirrhosis (AC) but not in heavy drinkers without liver disease^[9]^. Among patients with AC, recent alcohol consumption increased peripheral NK-cell numbers but reduced cytolytic ability^[9]^. Patients with sAH also have reduced cytolytic activity in both NK-cell and CD8 T cells^[10]^. Others have reported that NK cells were activated and had reduced cytolytic activity in patients with obesity, while patients with non-alcoholic steatohepatitis (NASH) had normal NK-cell function^[18,19]^. Together, these data suggest that alcohol consumption and ALD impair cytotoxic cell function, but how disrupted metabolic pathways lead to loss of cellular function is unknown.

Fatty liver diseases, as well as alcohol consumption, are characterized by impaired gut barrier function resulting in increased circulating concentrations of LPS. Importantly, LPS concentrations in patients with ALD and NAFLD are much lower than in septic conditions, ranging from 50–150 pg/ml^[20,21]^. Similarly, an acute binge of alcohol results in mild endotoxemia, with peripheral LPS concentrations of 70–100 pg/ml^[22]^. Exposure of immune cells to this chronic, low concentration of LPS likely has a profound impact on immune function.

In this work, we hypothesize that perturbation in the coordinated regulation of essential genes involved in cytotoxic cell function contributes to impaired cytotoxic function in sAH, a stage of ALD particularly prone to infection. Using scRNA-seq, CLR genes were dysregulated in CD8 T cells, while key genes involved in cytolytic granules were dysregulated in NK cells in sAH. Furthermore, flow cytometric analysis revealed a loss of key components of cytolytic granules in sAH. Together, these data provide a clear mechanism for the reduced cytolytic ability of NK cells in sAH.

## METHODS

### Selection of patients with liver disease and healthy controls

Enrolled patients had confirmed diagnoses of AH or NASH by clinicians at the Cleveland Clinic based on medical history, physical examination, and laboratory results, according to the guidelines of the American College of Gastroenterology (https://gi.org/clinical-guidelines/). Moderate and severe AH were determined by Model for End-Stage Liver Disease (MELD) score, where moderate was a MELD less than 20 and severe greater than 20. Healthy controls and heavy drinkers with an AUDIT score greater than > 16 were recruited from the Clinical Research Unit at the Cleveland Clinic or MetroHealth Hospital. Detailed clinical information on the patients used for single-cell analysis was reported by Kim *et al*. in 2020^[23]^, and clinical data for patient samples used for flow cytometry are presented in Supplementary Table 1.

### Study approval

The study protocol was approved by the institutional review board for the Protection of Human Subjects in Research at the Cleveland Clinic and University Hospitals, Cleveland, IRB: 08–361 (Nagy) and 17–718, 19–1041 (Dasarathy). All methods were performed in accordance with the internal review board’s guidelines and regulations, and written informed consent was obtained from all subjects.

### Isolation of human PBMCs

PBMCs were isolated from human blood as previously described^[23,24]^. Isolation of mononuclear cells was performed by density gradient centrifugation on Ficoll-Paque PLUS (GE Healthcare, Uppsala, Sweden). Cells were cryo-preserved in freezing media (50% culture media, 40% FBS, 10% DMSO) at a concentration of 1.5 × 10^6^ cells/mL. For experiments, cells were thawed at 37 °C, then added slowly to 8 mL of warm culture media. After centrifugation at 400 × g for 8 min at 20 °C, cells were resuspended and cultured in 96-well plates in a humidified atmosphere (5% CO_2_, 37 °C). After 18 h, cells were washed with media and challenged with 100 pg/ml LPS [Escherichia coli strain L2654 (O26:B6) Lot 120M4028, Sigma-Aldrich for 24 h]. This low concentration of LPS simulates the mild endotoxemia observed after binge consumption of alcohol and sAH^[21–23]^, as well as in NAFLD^[20]^.

### Clustering and differential expression

Sequencing data were aligned to the human genome (GRC38, release 93) using CellRanger (v3.0.2). All gene expression and clustering analyses were performed using Seurat (3.1.1) as previously described^[23,25]^. Briefly, all samples were first normalized using SCTransform and then filtered to remove low-quality cells (nFeature_RNA < 4000, nFeature_RNA > 200, percent.mt < 20, which removes doublets, cells with low reads, and cells with high mitochondrial content)^[26]^. All samples with the PBMC data from Ding *et al*. and Stuart *et al*., which contains data from eight different single-cell RNA sequencing platforms, were combined using the PrepSCTIntegration and FindIntegrationAnchors functions to find common anchor genes in all samples for all cell types, and then integrated using the IntegrateData function, with all normalizations using the SCT transformed data^[25,27]^. Clustering was performed using RunPCA and RunUMAP, and clusters were identified using FindNeighbors and FindClusters. Clusters were then identified using the publically available PBMC data^[25,27]^. Differentially expressed genes were measured using Seurat FindMarkers function (log-ratio) with paired, age-matched samples used as covariates.

### bigSCale2

For correlation analyses, bigSCale2 was used to calculate correlations using the Z-score algorithm, as previously described^[23,28]^. Single-cell RNA-seq data is inherently noisy, so the bigscale2 package utilizes a novel algorithm for measuring correlation coefficients by measuring differential gene expression between many smaller groups of cells. For these analyses, only NK cells were used. The gene set used for these figures were all genes predicted to be differentially expressed in NK cells either between sAH and HC, or in response to LPS in either patient category. We also added PRF1 to this gene list, as it is not statistically differentially expressed in any condition but was of particular interest as a component of the cytolytic granule. Genes were then filtered for low expression using the criteria that bigSCale2 was unable to determine a correlation coefficient. Heatmaps of the correlation coefficients were made with only the top 2.5% and bottom 2.5% of all correlation coefficients shown to remove noise and isolate the most important correlations.

### Flow cytometry

Cryopreserved cells were thawed and washed to remove excess freezing media. Cells were treated with DNAse to reduce clumping. Recovery from cryopreservation after DNAse was between 40%−70% and viability was > 95%. After washing, cells were then resuspended in FACS buffer (PBS, 1% BSA, 0.05% sodium azide) at a concentration of 1 × 10^6^ cells/mL and 200 μl were aliquoted into two wells of a 96-well U-bottom totaling ~4 × 10^5^ cells. As there were no data to do a prior power analysis, we performed an exploratory study using cryopreserved PBMCs from patients with severe AH (sAH, *n* = 8), moderate AH (mAH, *n* = 8), heavy drinkers (HD, *n* = 7), NASH (*n* = 8), and healthy controls (HC, *n* = 7). Cells were centrifuged at 830 g for 4 min and resuspended in 50 μl FACS buffer containing UV viability dye (L23105, Thermofisher, Waltham, MA). Cells were washed with FACS buffer, centrifuged again, then resuspended in Human TruStain FcX^™^ (Fc Receptor Blocking Solution, Biolegend, San Diego, CA) and incubated for 15 min at room temperature, according to manufacturer’s instructions. After blocking, cells were stained with extracellular fluorochrome-conjugated antibodies: PerCP-CD3 (clone SK7, Biolegend), BV510-CD8 (clone SK1, Biolegend), PE-Cy7-CCR7 (clone G043H7, Biolegend), PE/Dazzle-CD45RA (clone HI100, Biolegend), BV711-CD56 (clone HCD56, Biolegend) for 30 min at 4 °C in the dark. Cells were washed twice with FACS buffer and centrifuged at 830 g for 4 min. Cells were fixed and permeabilized using the Foxp3/Transcription Factor Staining Buffer Set (00-5523-00, eBioscience, Sand Diego, CA) at 4 °C for 30 min, according to the manufacturer’s instructions. Cells were then washed twice and centrifuged at 830 g for 5 min. Cells were resuspended in the blocking buffer again for 15 min at room temperature in the dark. After blocking, cells were stained with intracellular fluorochrome-conjugated antibodies: FITC-GZMA (clone CB9, Biolegend), PacBlue-GZMB (clone GB11, Biolegend), and APCFire-PRF1 (clone B-D48, Biolegend) for 30 min at 4 °C in the dark. Cells were then washed and resuspended in 100 uL of FACS buffer. Data were collected on a Cytek Aurora (Cytek, Fremont, CA) and analyzed using FlowJo software (Tree Star, Inc., Ashland, OR).

### Statistical analysis for flow cytometry

Data were analyzed by Analysis of Variance using a general linear model and least square means test for comparisons between groups (SAS, Carey, IN). The normal distribution of the data was assessed using the Shapiro-Wilk test and data were log-transformed, if necessary, to obtain a normal distribution.

Statistical difference between groups was determined at *P* < 0.05 and indicated by asterisks.

## RESULTS

### Cytotoxic cells in sAH have uncoordinated gene expressions

In a previous study using single-cell RNA-seq (scRNA-seq), we investigated the impact of challenging PBMCs from sAH (*n* = 4) patients and age-matched healthy controls (HC, *n* = 4) with and without 100 pg/mL LPS on the function of monocytes. Here we extend our analysis to NK and CD8-T cells. Clustering analysis identified one cluster of NK cells and four clusters of CD8 T cells, which we identified as naïve, memory, and two groups of effector cells. At baseline, many genes were upregulated in NK cells and effector CD8 T cells in sAH, while memory and naïve CD8 T cells had few changes [Figure 1B]. Cathepsins and exocytosis genes were among the most upregulated in cytotoxic cells. The only gene downregulated in both NK cells and effector CD8 T cells was Granulysin (GNLY) [Figure 1C]. Also downregulated in CD8 T cells from sAH were two KLRs, KLRG1 and KLRC2 [Figure 1C and D]^[29]^. KLRB1 and KLRC1 were not differentially expressed.

Downregulation of granulysin gene expression suggests a loss of cytolytic granule function, but no other major components of cytolytic granules were downregulated. While overall expression appeared similar between HC and sAH, we hypothesized that in sAH, coordinated expression of these genes, which are involved in cell maturation, might be lost within individual cells^[16]^. Indeed, the expression of cytolytic granules and other critical NK-cell genes were tightly correlated in HC [Figure 1E], but in sAH, the expression of most of these genes was no longer correlated. Thus, scRNA-seq revealed a loss of coordination and dysfunctional maturation of NK cells in sAH [Figure 1F]. Interestingly, while critical NK-cell genes lost coordinated expression, another set of genes, including cathepsins and exocytosis genes, were highly correlated in sAH [Figure 1F].

### LPS activates more robust inflammatory responses in sAH NK cells

**Figure 2.**
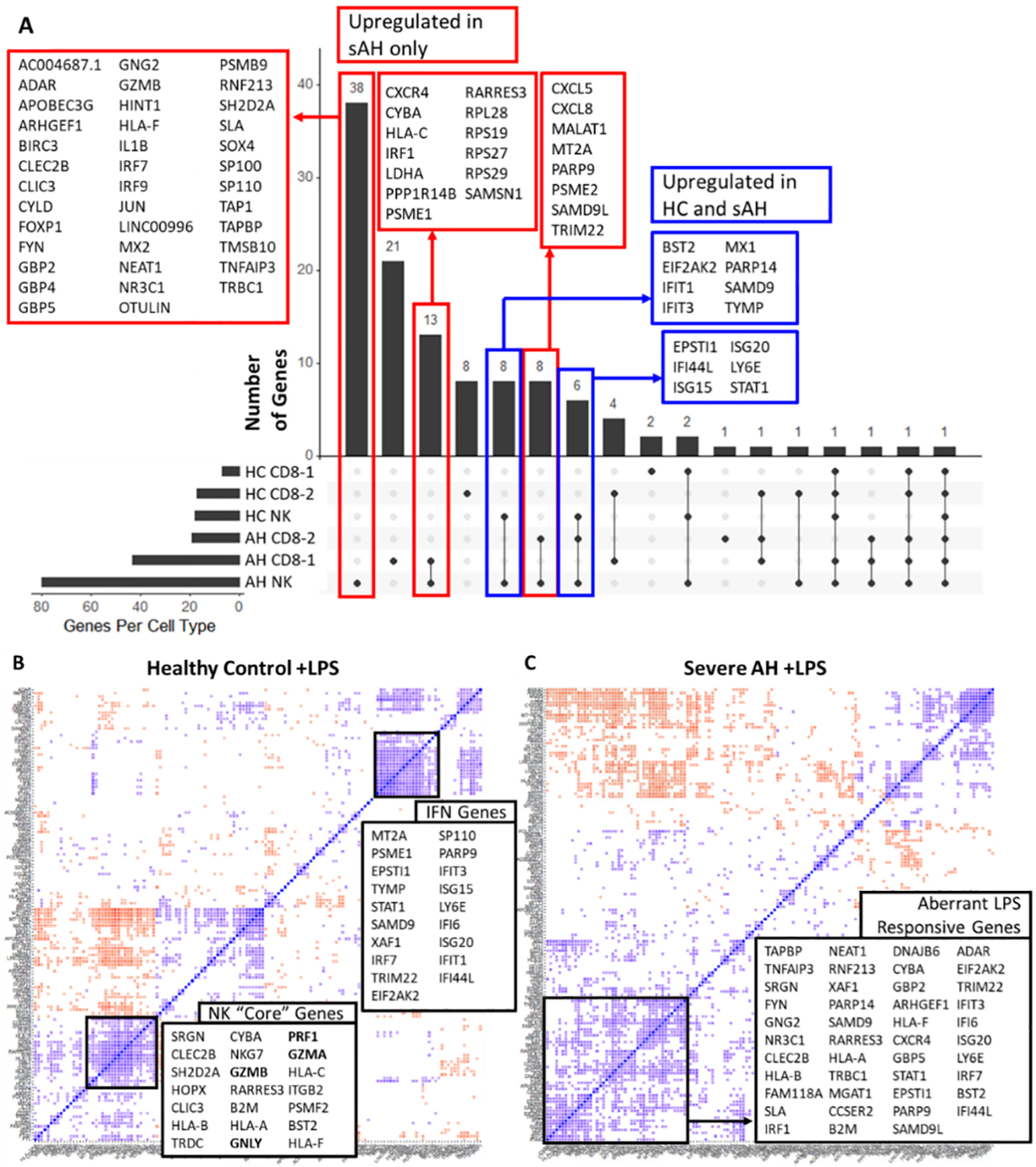
LPS upregulates additional pro-inflammatory genes in sAH. (A) UpSet plot summarizing the LPS responsive genes in NK cells and 2 clusters of effector CD8 T cells from sAH and HC. Bars indicate the number of genes upregulated in response to LPS. Black dots indicate intersections of the data, or in other words, genes upregulated in multiple cell types. Red boxes are genes upregulated only in sAH cytotoxic cells. Blue boxes are genes upregulated in sAH and HC cytotoxic cells. (B and C) Correlation analysis to see how genes in NK cells are coordinately expressed in response to LPS for healthy control (B) and sAH (C). Blue squares indicate genes with highly correlated expression, while red squares indicate anti-correlated expression. Cytolytic granule genes are in bold. sAH: Severe alcohol-associated hepatitis; HC: healthy controls.

We hypothesized that one consequence of diminished cytotoxic cell maturation and cytolytic granule dysfunction would be reduced IFNγ signaling [Figure 1A]^[11]^. However, both NK- and CD8 T cells from sAH and HC expressed interferon-associated genes in response to LPS, although the IFNγ gene was undetectable. These results suggest normal interferon signaling in sAH, even though cell maturation is perturbed (Figure 2A, Blue Boxes). Furthermore, LPS also upregulated numerous chemokines, cytokines and other inflammatory genes in cytotoxic cells from patients with sAH, but not in HC (Figure 2A, Red Boxes)^[11]^.

When we examined coordinate expression in HC, three clusters of genes were highly co-regulated in NK cells in response to LPS [Figure 2B]. One cluster contained almost all of the core NK-cell genes observed at baseline, including cytolytic granule genes [Figure 2A and B]. Interferon-associated genes were correlated with each other in a separate cluster. These results imply that, in HC, NK cells have independent regulation of cytolytic granules and interferon-responsive genes. However, in sAH, cytolytic granule genes remained dysregulated after LPS, but interferon genes and other NK-cell genes were highly correlated [Figure 2C]. These results are consistent with the hypothesis that in sAH, NK cells are not properly matured, leading to dysregulation of core genes, including cytolytic granules, while inflammatory gene expression is highly coordinated and prioritized.

### NK cells in sAH have diminished cytotoxic granules

**Figure 3.**
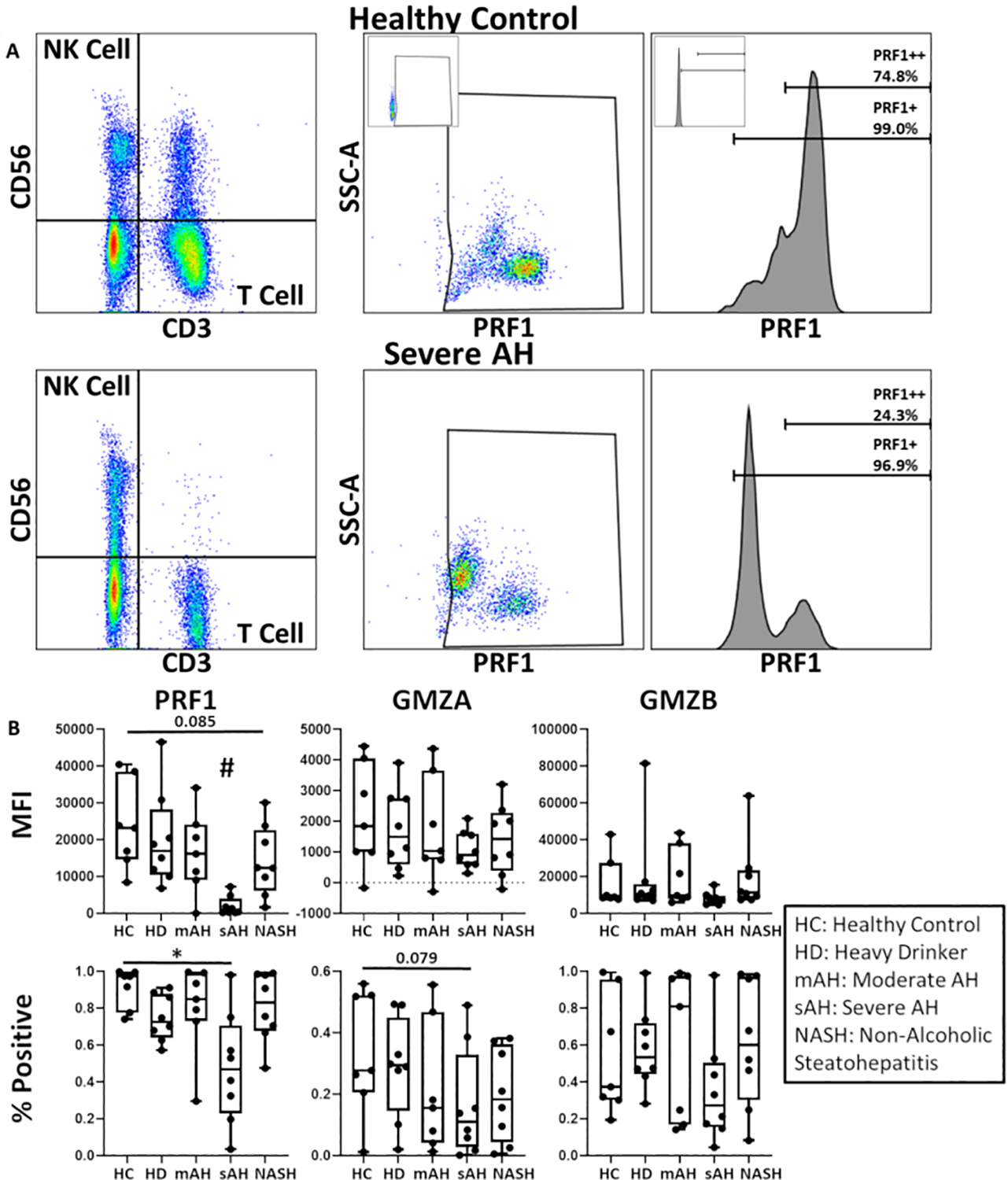
NK-cell have reduced cytolytic granules in sAH. Flow cytometry of PBMCs from different metabolic liver disease patients to assess protein expression of granzyme B and perforin in NK cells and CD8 T cells. (A) NK cells, NKT-cells, and T-cells were defined by the expression of CD56 (NK cells), CD3 (T-cells), or both (NKT-cells). CD8 T cells were identified by the expression of CD8. Then perforin (PRF1), granzyme A (GZMA), and granzyme B (GZMB) were measured by median fluorescence intensity (MFI) and % positive cells. (B) MFI and % positive cells were measured for PRF1, GZMA, and GZMB within each disease. Error bars indicate standard deviation, * = *P* < 0.05, ^#^ = *P* < 0.05 compared to all groups. sAH: Severe alcohol-associated hepatitis.

The scRNA-seq data suggest dysfunctional cytolytic granules in NK cells in sAH. We hypothesized that in sAH, immature cytolytic granules are observed in NK cells, which result in NK cells lacking one or more components. To test this hypothesis, we used intracellular flow cytometry to measure the expression of GZMA, GZMB, and PRF1 protein in NK cells and effector CD8 T cells [Figure 3A]^[16]^. PBMCs from patients with sAH (*n* = 8), moderate AH (mAH, *n* = 8), heavy drinkers (HD, *n* = 7), NASH (*n* = 8), and HC (*n* = 7) were analyzed in order to assess cytolytic granule dysfunction in different stages of AH [Figure 3B, Supplementary Table 1 and Supplementary Figure 1]. In patients with sAH, we observed a stark decrease in PRF1 protein in NK cells and fewer PRF1^+^ and GZMA^+^ cells in sAH. Interestingly, no significant change in any of these three proteins was observed in HD or mAH, suggesting that loss of PRF1 is a phenotype of severe disease. We also find no loss of cytolytic granules among the patients with NASH, similar to what has been shown in the literature^[19]^. In conclusion, the loss of coordinated expression between cytolytic granule genes led to decreased protein levels of PRF1 in sAH, which would render these cells unable to lyse target cells.

## DISCUSSION

In this study, we utilize bioinformatic analyses of scRNA-seq data and multi-panel intracellular flow cytometry to better understand gene regulation and cellular dysfunction among cytotoxic cells in sAH. We found that NK cells have both disrupted regulation of cytolytic granule genes and loss of cytolytic proteins in sAH, which is significant because these genes are required for mature, functional cells^[16]^. In response to LPS, genes associated with IFNγ signaling were upregulated in NK and CD8 T cells in both HC and sAH, as well as many pro-inflammatory cytokines in sAH.

Patients with sAH experience exacerbated inflammation in both the liver and peripheral immune systems. Recent studies found patients to have increased numbers of peripheral myeloid cells^[30]^, increased sensitivity to LPS^[23,31]^, and increased infiltration of peripheral monocytes into the liver^[32]^. However, less is known about cytotoxic cells in sAH and their response to LPS, even though these cells play a critical role in regulating, removing, and communicating with other immune cells. Importantly, cytotoxic cells are critical to preventing bacterial and viral infections, a serious and often fatal complication of metabolic liver diseases.

Cytotoxic cells have two major functions: killing activated or infected target cells and IFNγ signaling. We find that both of these functions are altered in sAH. First, we observed decreased expression of cell-cell receptors in CD8 T cells: KLRC2 and KLRG1. KLRC2 is an inhibitory receptor, similar to checkpoint inhibition, while KLRG1 is a marker for effector CD8 T cells^[11,33]^. Downregulation of these genes indicates a decreased ability to lower inflammatory responses and loss of cell maturity.

Previous studies found that NK cells in sAH have diminished cytolytic functions; *in vitro* studies found NK cells from ALD patients had reduced cytolytic capacity by an undetermined mechanism^[9,10]^. We hypothesize that the uncoordinated expression of mRNA is due to dysregulated NK-cell maturation, which would lead to fewer cytolytic granules. However, the upregulation of proteolytic and exocytosis pathways suggests that it is the lack of cytolytic granules, rather than impaired secretion of granules, that impacts function^[34]^. Loss of mature cytolytic granules makes these NK cells unable to kill target cells, thus lowering resistance to infection.

While cytotoxic cells may be dysfunctional in terms of targeted cell killing in sAH, many more pro-inflammatory genes were upregulated compared to HC in response to LPS. Interestingly, our previous study of monocyte populations in patients with sAH also revealed aberrant regulation of diverse pro-inflammatory and interferon-associated genes^[23]^. For example, one CD14^+^ monocyte population with anti-viral and anti-inflammatory properties in HC shifted to a pro-inflammatory phenotype lacking anti-viral expression in sAH. Taken together, these data indicate a profound shift in the expression of interferons and anti-viral genes in both monocytes, NK cells and CD8 T cells in sAH.

While the scRNA-seq data focused on gene expression changes in sAH compared to HC, using flow cytometry, we were able to expand our study to look at heavy drinkers, moderate AH, and NASH. Thus, we were able to conclude that NK-cell dysfunction, via loss of cytolytic granules, is specific to sAH, and not a symptom of alcohol consumption or metabolic disorder. This is an important point because recent studies have found that sAH is unique among the spectrum of liver diseases in that many metabolic functions are significantly perturbed, including lipid metabolism^[35]^, bile acid production^[36]^, and microbial metabolites^[37]^. Together, these results suggest that sAH is a severe metabolic disorder that damages many organs and cells in the body, including the immune system.

From our data, we cannot conclude whether NK-cell dysfunction is a cause of sAH or just another complication in the disease progression and metabolic dysfunction. NK-cell dysfunction and loss of cell-cell communication between NK cells and monocytes likely contribute to the increased pro-inflammatory environment observed in sAH. We hypothesize that cell-cell communication via C-type lectin receptors provides critical feedback between NK cells and monocytes, and loss of these pathways might result in a lack of proper NK-cell maturation and targeted killing of activated monocytes.

## CONCLUSION

In conclusion, previous reports found that NK cells are dysfunctional and unable to kill target cells^[10]^. Mechanistically, we show that sAH is associated with a loss of coordinate expression of genes and proteins required for the formation of cytolytic granules. Without cytolytic granules, NK cells would be unable to perform their most critical function of killing target cells, which could contribute to an increased risk of infection and poor removal of stressed and potentially malignant cells, increasing the risk for cancers.

Instead, NK cells and CD8 T cells are far more pro-inflammatory in sAH, making them possible contributors to exacerbated inflammation in this disease.

## Supplementary Material

Supplementary Info

## Data Availability

The scRNA-seq data for this study can be found at National Center for Biotechnology Information. Gene Expression Omnibus under accession number PRJNA596980. All scripts used for analyses and differential expression results for all cell types can be found at the author’s github (github/atomadam2).
