# Supplementary material for: Diminished function of cytotoxic T- and NK- cells in severe alcohol-associated hepatitis
Source: Metab Target Organ Damage. Author manuscript; Available in PMC 2024 Aug 15. (PMC11326509; doi:10.20517/mtod.2022.13)
Supplement: Supplementary Info [file NIHMS2014954-supplement-Supplementary_Info.pdf]

Supplementary Materials: Diminished function of cytotoxic T- and NK- cells in severe alcohol-associated hepatitis

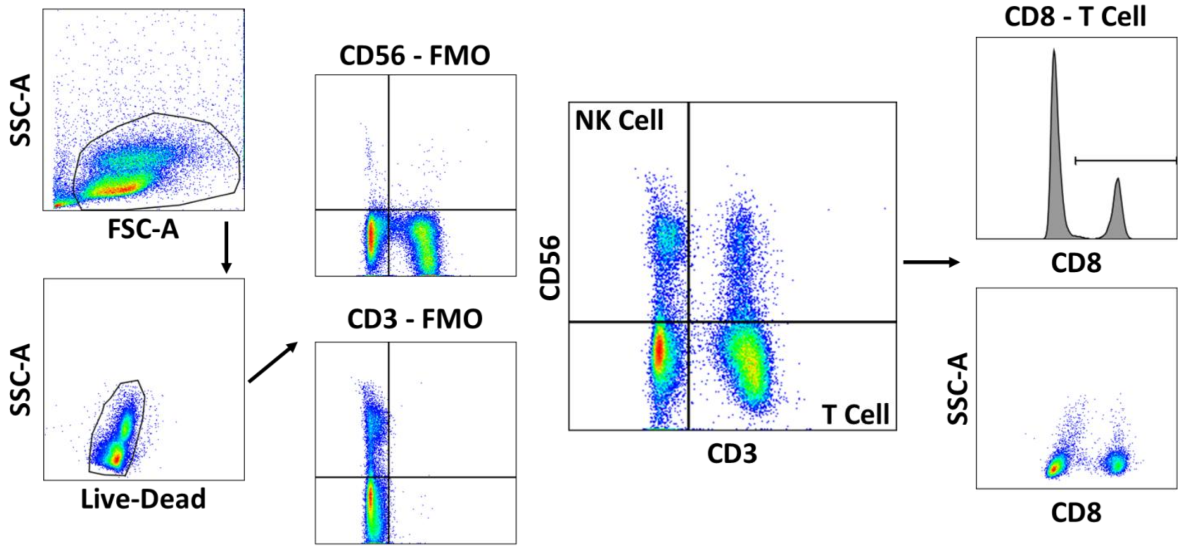

Supplementary Figure 1. Gating strategy for flow cytometry.

Supplementary Table 1: Clinical data for patient samples used in flow cytometry experiment

Median (IQR)	**Healthy Control (HC), n=7**	**Heavy Drinker (HD), n=8**	**Moderate AH (mAH), n=7**	**Severe AH (sAH), n=8**	**Non-Alcohol Steatohepatitis (NASH), n=8**
Male n (%)	3 (43)	4 (50)	4 (57)	3 (38)	4 (50)
Age (years)	42 (35.5-57)	54 (39-61)	54 (48-56)	54 (40.75-57.5)	49.5 (40.75-62.25)
AST (U/L)	18 (16-20)	29.5 (24.75-42.75)	65 (46.5-85)	112.5 (61.25-152)	39.5 (26-90.5)
ALT (U/L)	17 (12-20)	27.5 (23.75-33.75)	43 (25.5-63.5)	45 (34-65.25)	63 (35.5-85.25)
Serum Albumin (g/dL)	4.3 (4.3-4.3)	4.55 (4.475-4.65)	3.6 (2.9-4.05)	2.95 (2.7-3.125)	4.55 (4.4-4.925)
Serum Creatinine (mg/c	1.02 (0.85-1.04)	0.8 (0.77-0.89)	0.73 (0.65-0.77)	1.08 (0.8625-1.6825)	0.935 (0.735-1.1275)
Serum Bilirubin (mg/dL	0.5 (0.3-0.6)	0.4 (0.325-0.7)	1.2 (0.25-4.7)	23.25 (15.2-32.775)	0.45 (0.325-0.5)
MELD Score	(-)	(-)	7 (6.5-19)	30.5 (28.25-33)	6.5 (1.5-8.5)
